# The Kinetics of Chitosan Degradation in Organic Acid Solutions

**DOI:** 10.3390/md19050236

**Published:** 2021-04-22

**Authors:** Dominik Sikorski, Karolina Gzyra-Jagieła, Zbigniew Draczyński

**Affiliations:** 1 Institute of Textile Materials and Polymer Composites, Lodz University of Technology, Zeromskiego 116, 90-924 Łódź, Poland; k.jagiela@ibwch.lodz.pl (K.G.-J.); zbigniew.draczynski@p.lodz.pl (Z.D.); 2 Łukasiewicz Research Network—Institute Biopolymers and Chemical Fibres 19/27 M, Skłodowskiej-Curie Street, 90-570 Łódź, Poland

**Keywords:** chitosan, degradation, dissociation constant, viscosity

## Abstract

This paper presents a comparative study on chitosan degradation in organic acid solutions according to their different dissociation characteristics. More precisely, the aim of the study was to determine the kinetics of the degradation process depending on the different acid dissociation constants (pKa values). The scientists involved in chitosan to date have focused mainly on acetic acid solutions. Solutions of lactic, acetic, malic, and formic acids in concentrations of 3% wt. were used in this research. The progress of degradation was determined based on the intrinsic viscosity measurement, GPC/SEC chromatographic analysis, and their correlation. Changes in the viscosity parameters were performed at a temperature of 20 °C ± 1 °C and a timeframe of up to 168 h (7 days). The chemical structure and DDA of the initial chitosan were analyzed using 1H-NMR spectroscopy analysis. The results of this study can be considered of high importance for the purpose of electrospinning, production of micro- and nano-capsules for drug delivery, and other types of processing. Understanding the influence of the dissociation constant of the solvent on the kinetics of chitosan degradation will allow the selection of an appropriate medium, ensuring an effective and stable spinning process, in which the occurrence of polymer degradation is unfavorable.

## 1. Introduction

Chitin is the second most abundant polysaccharide in the natural environment, after cellulose. It is a structural polymer of marine invertebrate shells, insect skeletons, and fungal cell walls [1,2,3,4]. It can be found in the structure of sponges and corals [5,6]. The polymer was discovered by H. Braconnt in 1811, while the name was given by Odier, who, in 1823, discovered the same chemical compound in insects [7]. Chitin is a linear polysaccharide consisting mostly of 2-acetamido-2-deoxy-β-D-glucopyranose (GlcNAc) residues and partly of 2-amino-2-deoxy-β-D-glucopyranose residues (GlcN), linked together by a β- (1→4) glycosidic bond. Its chemical structure impedes its solubility in water and most organic solvents [8]. Due to this, numerous studies of its chemical modification are carried out in the field, leading to a soluble form in readily available solvents. There are known examples of introducing new functional groups on reactive hydroxyl groups present at the C-3 and C-4 atom of the sugar ring, which allow a polymer that is soluble in typical organic solvents to be obtained [9,10]. There is a significant body of literature on different types of chitin modification aiming at increasing its solubility in different organic and inorganic solvents [11,12]. However, these studies do not provide a comparative analysis conducted in the same conditions concerning the influence of the solvent dissociation constant on the kinetics of chitosan degradation.

Chitosan is a natural biopolymer obtained by enzymatic or chemical deacetylation of chitin and is considered its most well-known derivative. The increase in GlcN residues in the sugar chain results in solubility in dilute aqueous acid solutions (pH < 6) [13]. The degree of N-acetylation of chitin and chitosan is an important parameter in determining the physicochemical properties of the polymer, defined by the percentage of GlcNAc residues per 100 monomer units in the polymer chain [14]. When the degree of acetylation is above 50%, the polymer is called chitin, and, when the degree of acetylation is below 50%, the polymer is called chitosan [15,16]. While solubility of chitin is still a matter of interest of many research groups, factors affecting the solubility of chitin and chitosan described in the previous literature mainly concern the degree of polymer deacetylation, environmental pH, polymer molar mass, temperature, and acid ionic strength. Within those, the focus on the dissociation characteristics of acidic solvents and their impact on degradation kinetics is our main research objective.

However, chitin solubility does not depend only on its molar mass, but it is linked to the degree of acetylation that is related to the number of N-acetylamino groups in molecules [17]. Chitosan exhibits many favorable properties, e.g., the ability to create polycations in an acidic environment; its hydrophilic character; the ability to react with divalent metals, dyes and proteins; and the ability to create films [18,19]. This polymer is widely available in various forms: gels [20], aerogels [21], membranes [22], nanofibers [23], micro- and nano-particles [24], scaffolds [25], and sponges [26]. Due to its physicochemical properties and the possibility of using chitosan in various phases, such as solid, gel, and aqueous solutions, chitosan can be used for the production of new smart materials, such as nanocomposites and bio-nanocomposites [27,28]. Chitosan dissolves in aqueous acid solutions, such as acetic, citric, formic, hydrochloric, and lactic acid [29,30,31,32]. Most often, acetic acid with a concentration of up to 70% by weight is used to prepare chitosan spinning solutions for electrospinning, with the pH of spinning solutions being below 6.2 [33].

Chitosan is extremely sensitive to various chemical and physical factors, which consequently causes its degradation. Physical and chemical properties, as well as the rate of chitosan degradation processes, depend on the degree of deacetylation and the molar mass of this polymer. In the context of the use of chitosan in various biomaterials, the greatest threats are biodegradation, degradation by ultrasounds, thermal degradation, and photodegradation. Chitosan decomposition can take various forms: oxidative [34,35]; hydrolytic [36]—decomposition under the influence of water; thermal [37,38]; ultrasonic [39,40]; photodegradation (UV) [41]; or enzymatic [42]. Chitosan hydrolysis occurs mainly under the influence of acids [43,44]; thus, it is important to select the solvent providing the highest possible stability of the polymer and, by this, the stability of the electrospinning process. The great interest in this polymer results from the properties of the polymer: biocompatibility, biodegradability, nontoxicity, antimicrobial, and hydrating properties, which positively influence the wound healing process. Previous studies have shown that chitosan-based dressings can accelerate the repair of different tissues; facilitate the contraction of wounds; and regulate the secretion of inflammatory mediators, such as interleukin 8, prostaglandin E, interleukin 1 β, and others [45,46,47].

The research presents a comparative study on chitosan degradation in organic acid solutions according to its acid dissociation constant (pKa values). The results of the studies can be considered of high importance for the purpose of electrospinning, production of micro- and nano-capsules for drug delivery, and other types of processing.

## 2. Results

**Scheme 1:**
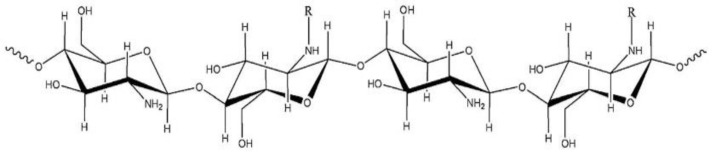
Structure of chitosan. G.A. Morris et al./Polymer Degradation and Stability 94 (2009) 1344–1348.

The values of intrinsic viscosity of diluted chitosan solutions subjected to the aging process in organic acids, differing in pKa and the average viscometric molar masses Mv determined on their basis, are presented in Table 1 and Scheme 1.

Table 1, presenting the viscosity average molar mass M_v_ and intrinsic viscosity, confirms the occurrence of degradation in all studied acids. The highest decrease in intrinsic viscosity occurs in the case of malic acid, while in the remaining acids, such as acetic, formic, and lactic acid, this impact is uniform with respect to the decrease in the average molar mass.

**Figure 1:**
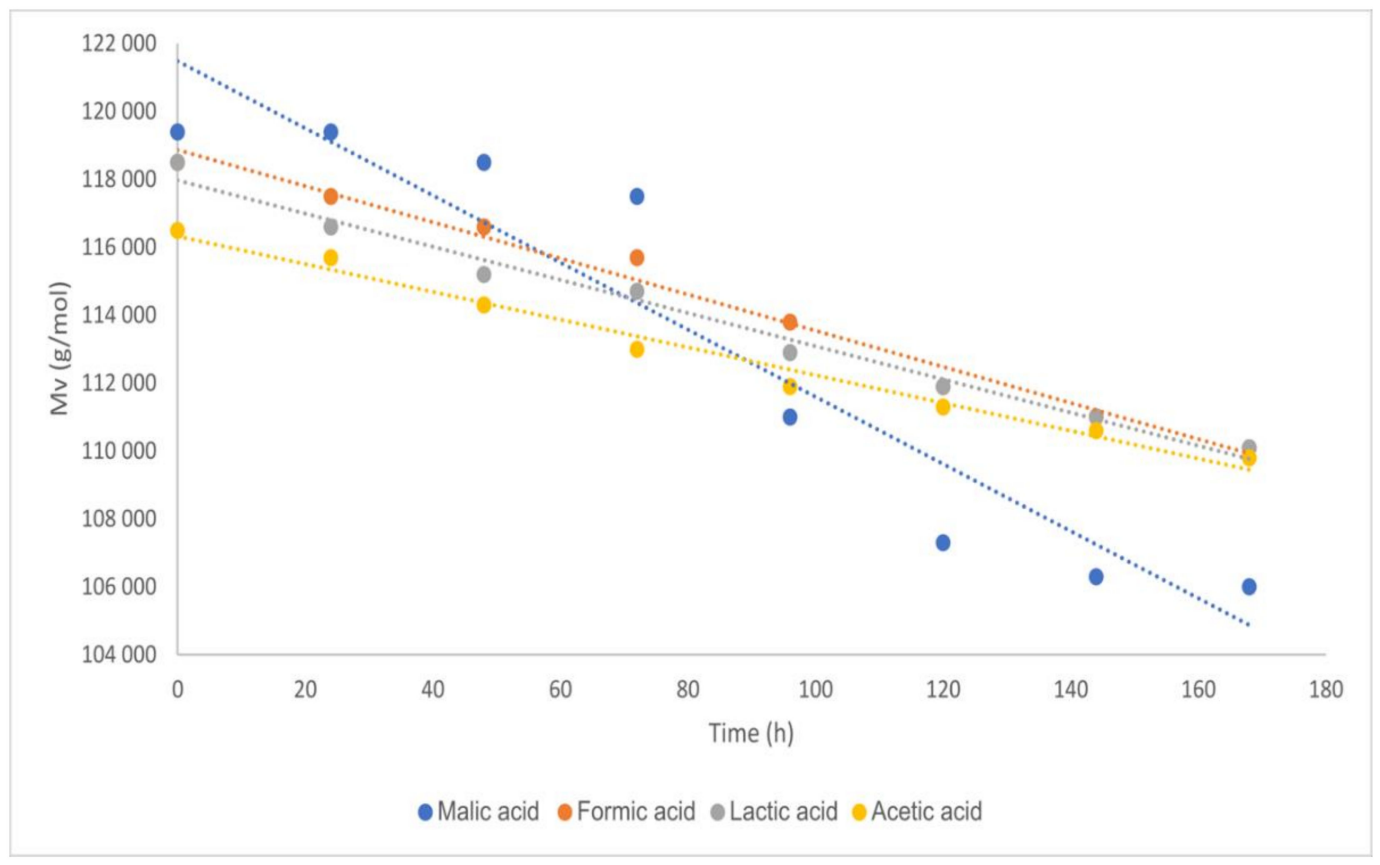
Chitosan M_v_ changes after the degradation time.

On the basis of the graphically presented relationship between M_v_ and degradation time, shown in Figure 1, visible changes are observed, indicating the ongoing polymer degradation process and Table 2 values of angular coefficient obtained from linear dependence of M_v_. The highest drop in M_v_ value over time, corresponding to a decrease of 11.22% in t = 168 h, is observed for chitosan treated with malic acid (slope value −150.24). In the case of formic acid, acetic acid, and lactic acid, the changes in M_v_ were not as rapid as in the case of malic acid (for acetic acid—5.75% decrease in *t* = 168; for formic acid and lactic acid—7.09%). Nevertheless, they also show a destructive effect on chitosan macromolecules. The next acid, in terms of degradation activity, is acetic acid followed by formic acid, while lactic acid is characterized by the least influence on M_v_ changes over time. The following ordering can be assigned (the degrading force): acetic acid→lactic acid→formic acid→malic acid.

### 2.1. Analysis of GPC/SEC Results

**Figure 2:**
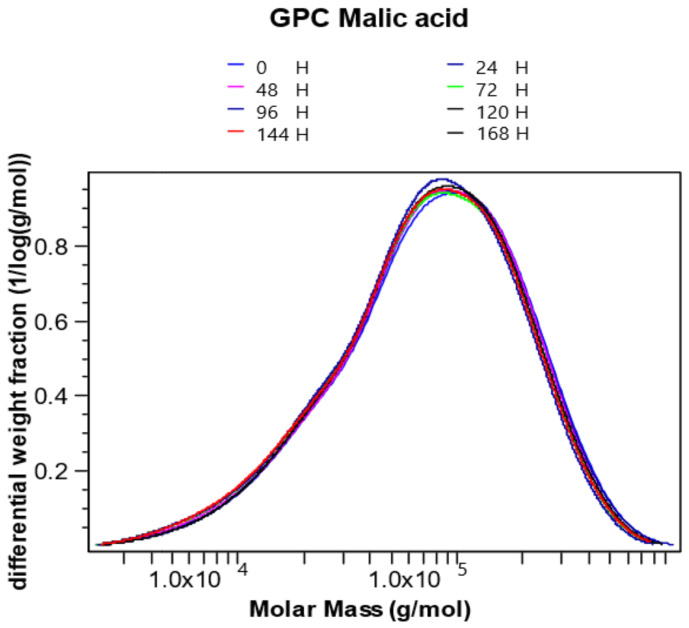
Molar mass distribution for chitosan in malic acid.

**Figure 3:**
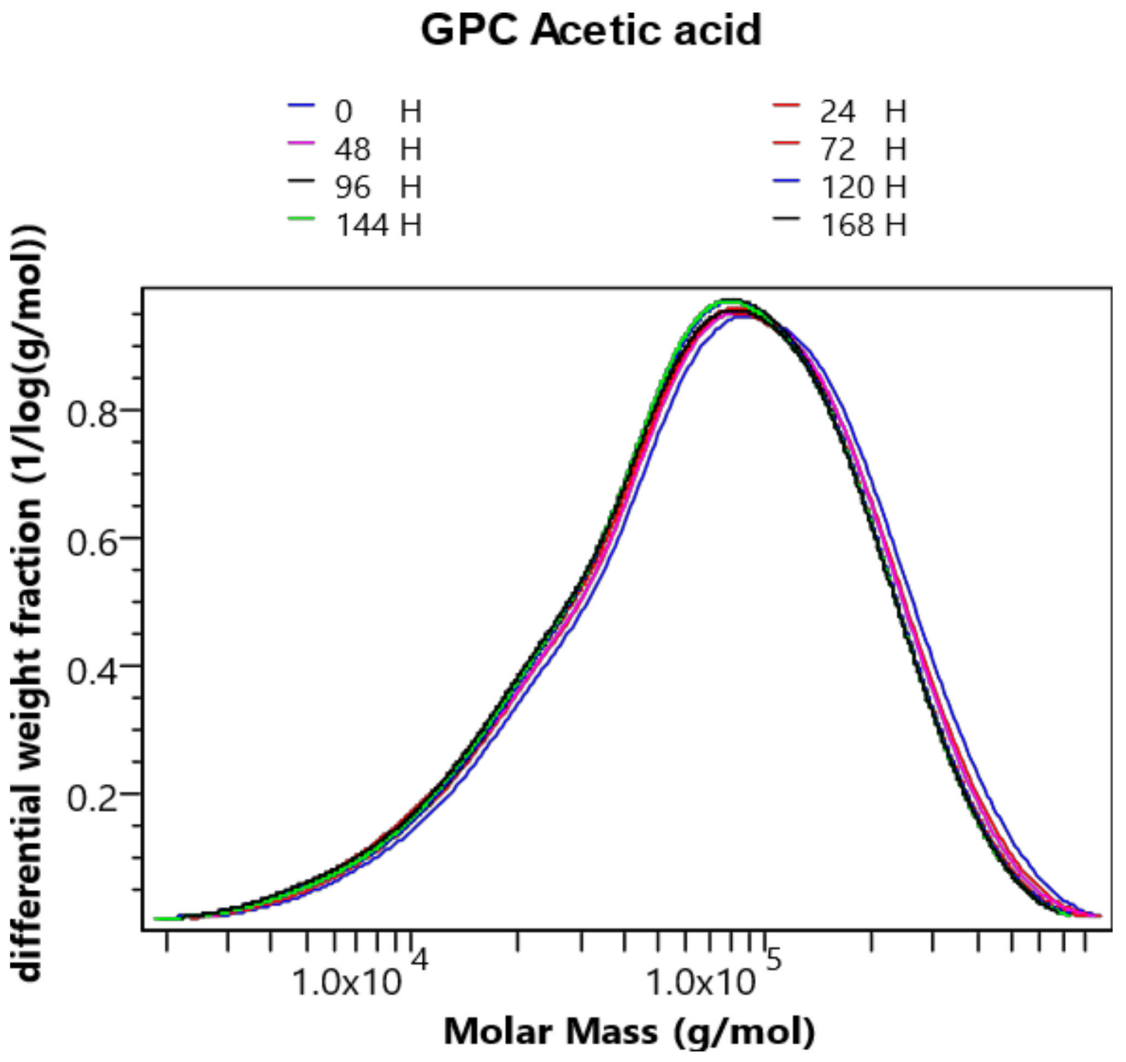
Molar mass distribution for chitosan in acetic acid.

**Figure 4:**
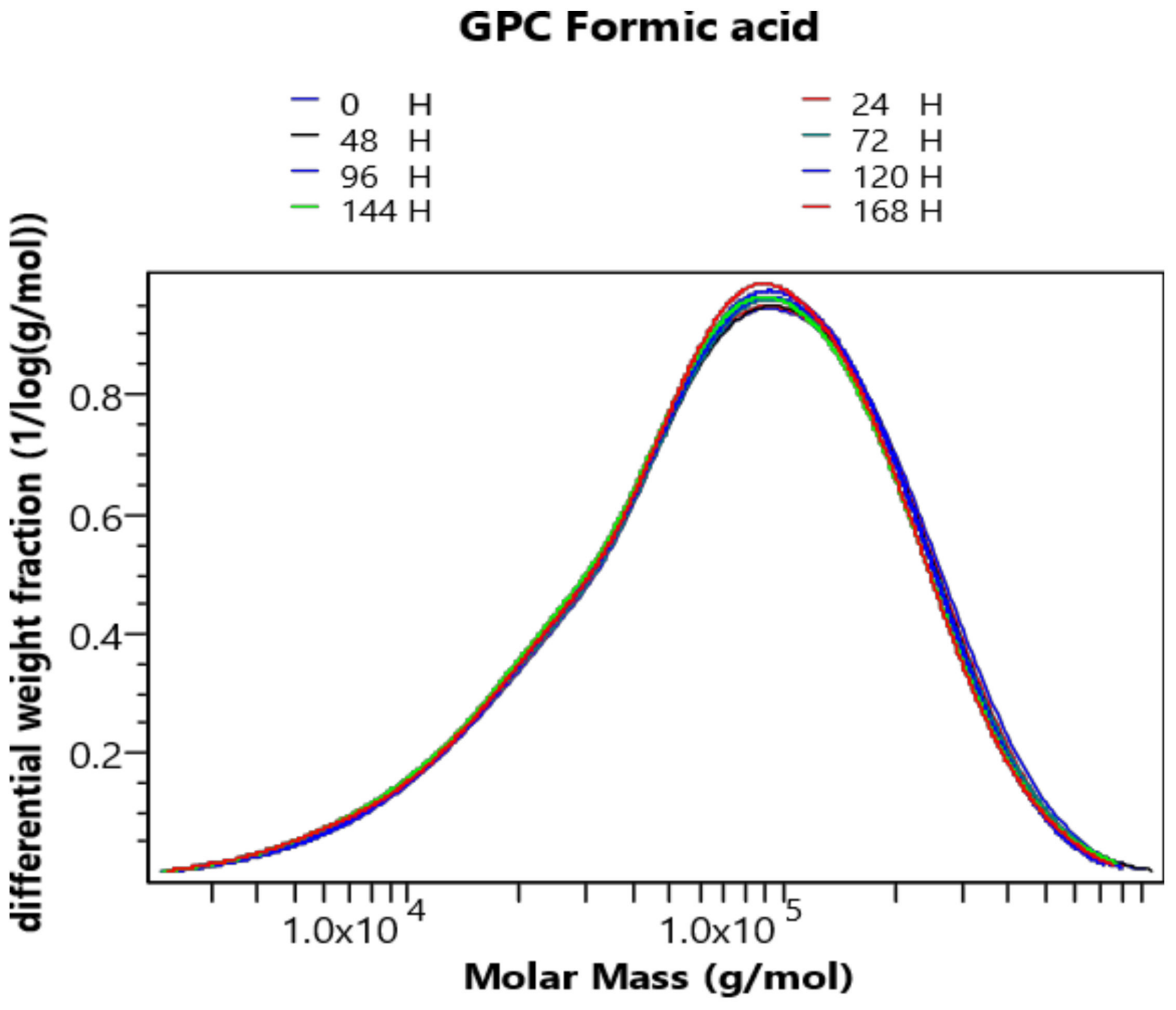
Molar mass distribution for chitosan in formic acid.

**Figure 5:**
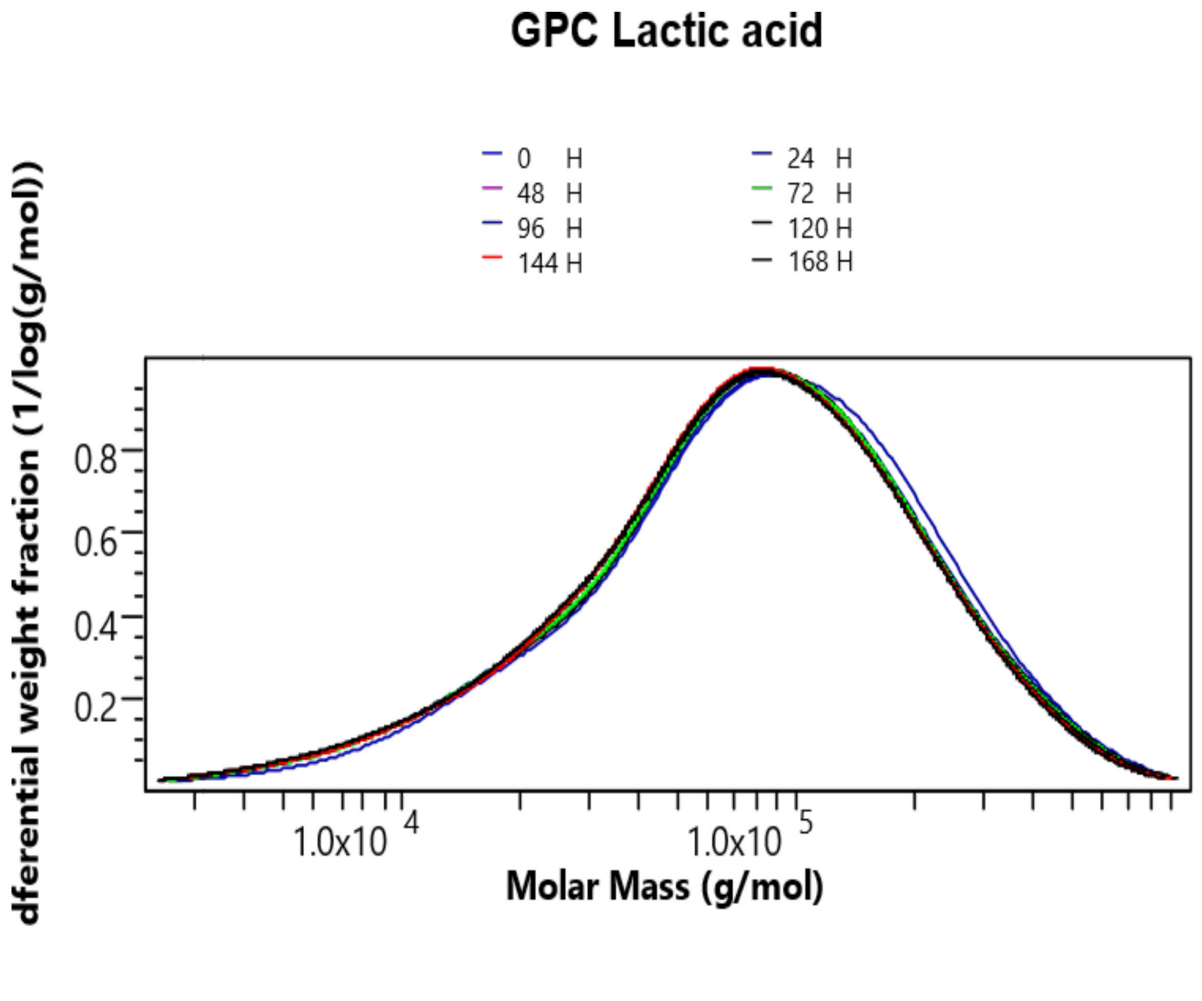
Molar mass distribution for chitosan in lactic acid.

**Figure 6:**
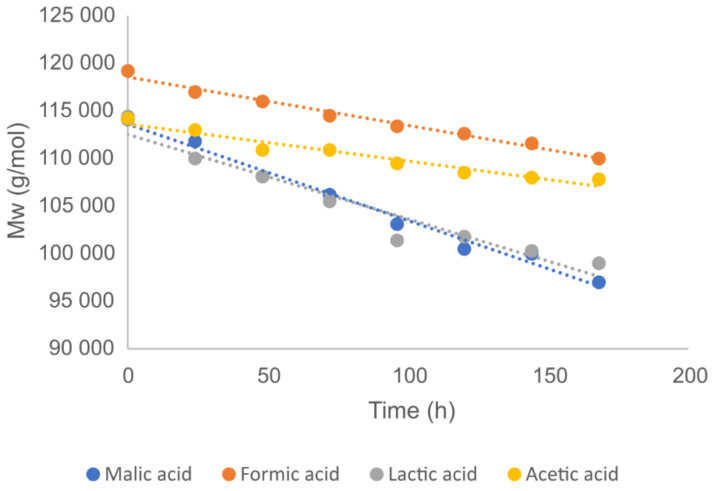
Chitosan M_w_ value changes after the degradation time.

**Figure 7:**
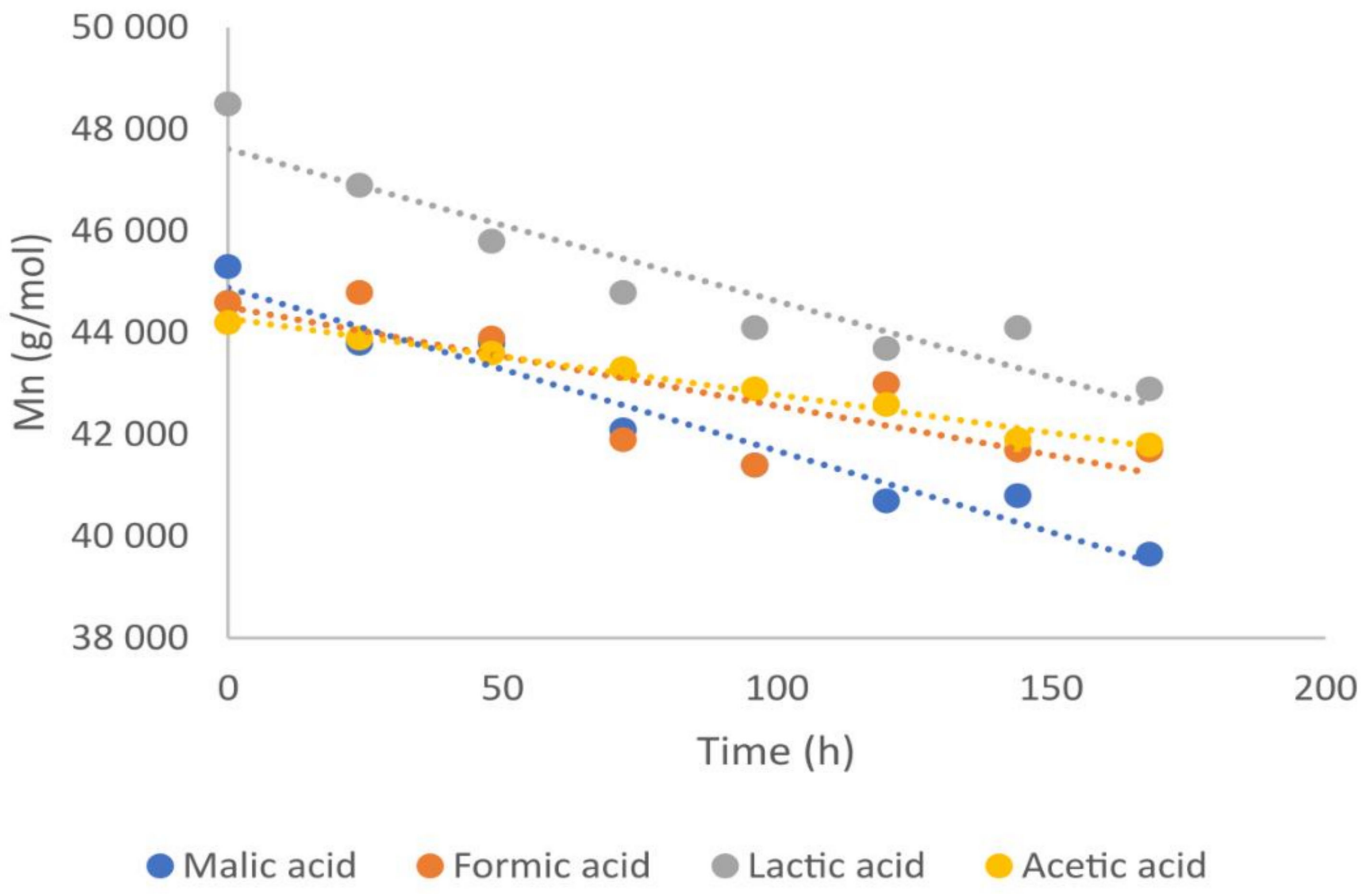
Chitosan M_n_ value changes after the degradation time.

**Figure 8:**
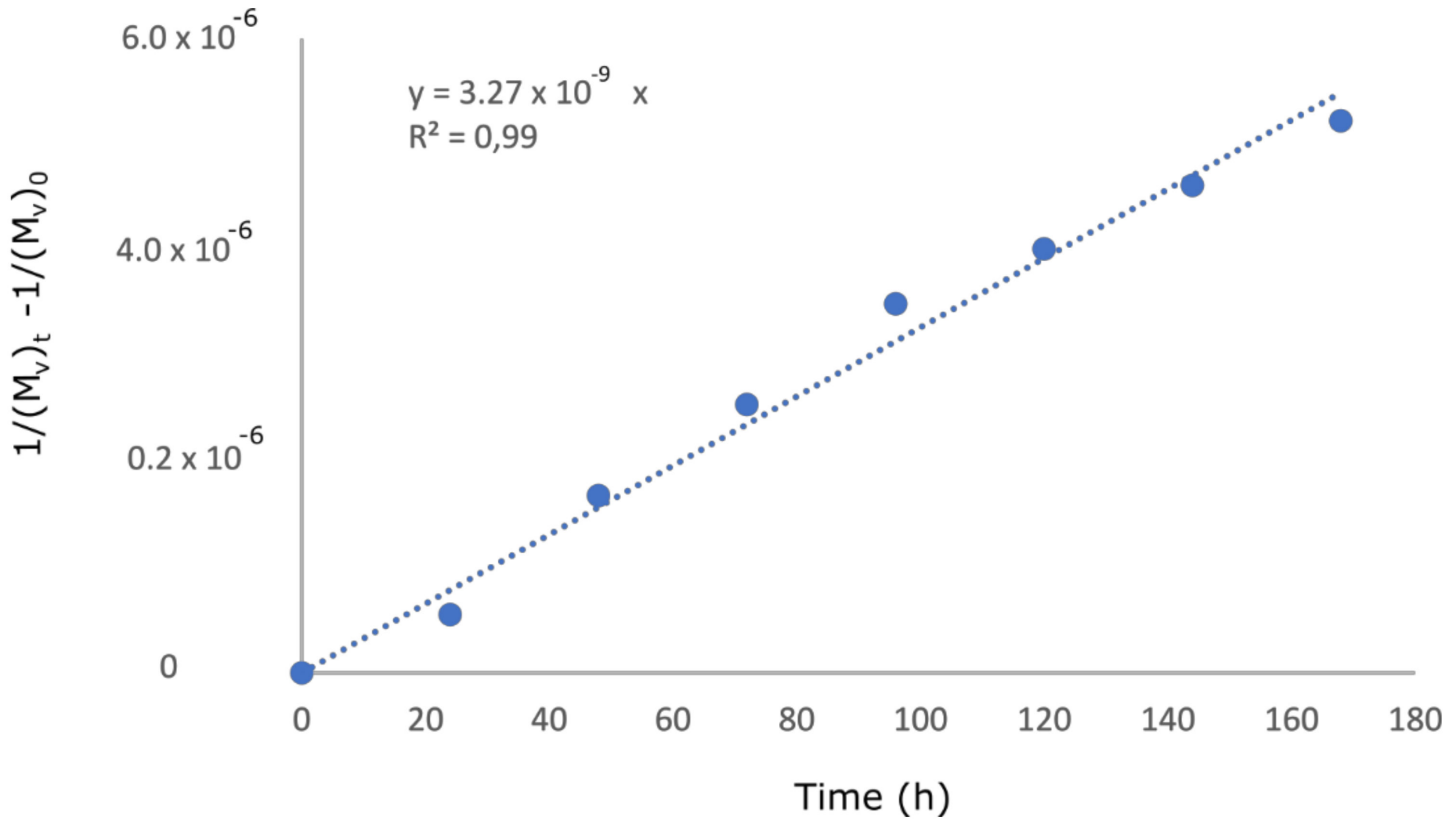
Degradation kinetics M_v_ values for chitosan in 3% of acetic acid at 20 °C.

On the basis of GPC/SEC chromatographic analysis, the following molecular parameters were determined: number average molar mass (M_n_), weight average molar mass (M_w_), polydispersity (Pd), and molar mass distribution (MMD) for chitosan samples subjected to the degradation process at 20 °C. The results are summarized in Table 3 and Figure 2, Figure 3, Figure 4, Figure 5, Figure 6, Figure 7 and Figure 8.

Table 3, presenting the molecular parameters determined by the GPC/SEC technique, confirms the occurrence of degradation in all studied acids. The highest decrease in Pd occurs in the case of lactic acid and malic acid. On the basis of the graphically presented relationship between M_w_ and degradation time, shown in (Figure 6, Table 4), the highest drop in M_w_ value over time is observed for chitosan treated with malic acid—slope value −101.49, followed by lactic acid—88.94, formic acid—50.94, and acetic acid—38.89%. A similar relationship is observed for Mn values (Figure 7, Table 5). The following ordering can be assigned (the degrading force) based on the GPC/SEC analysis: acetic acid→formic acid→lactic acid→malic acid.

### 2.2. Kinetics of Chitosan Degradation

The rate of depolymerization most often follows the mechanism of the primary relationship. In the case in which the mechanism complies with a first-order degradation, the kinetic equation is described by the following formula:

(1) $$\frac{1}{{\left(M_{w}\right)}_{t}}-\frac{1}{{\left(M_{w}\right)}_{t0}}=\frac{k}{M}\times t$$

By plotting the dependence $\frac{1}{{\left(M_{w}\right)}_{t}}-\frac{1}{{\left(M_{w}\right)}_{t0}}$ as a function of time, it is possible to conclude (on the basis of the linearity of the points) compliance with the degradation mechanism described by the first-order equation, as presented in [48]. Correlation values presented in Table 6, Table 7 and Table 8 additionally confirm the linearity of the function.

Figure 8 shows the first-order kinetic relationship for chitosan degradation in dilute acetic acid. As can be seen in the presented diagram, the degradation process is very well reflected by first-order kinetics.

**Figure 9:**
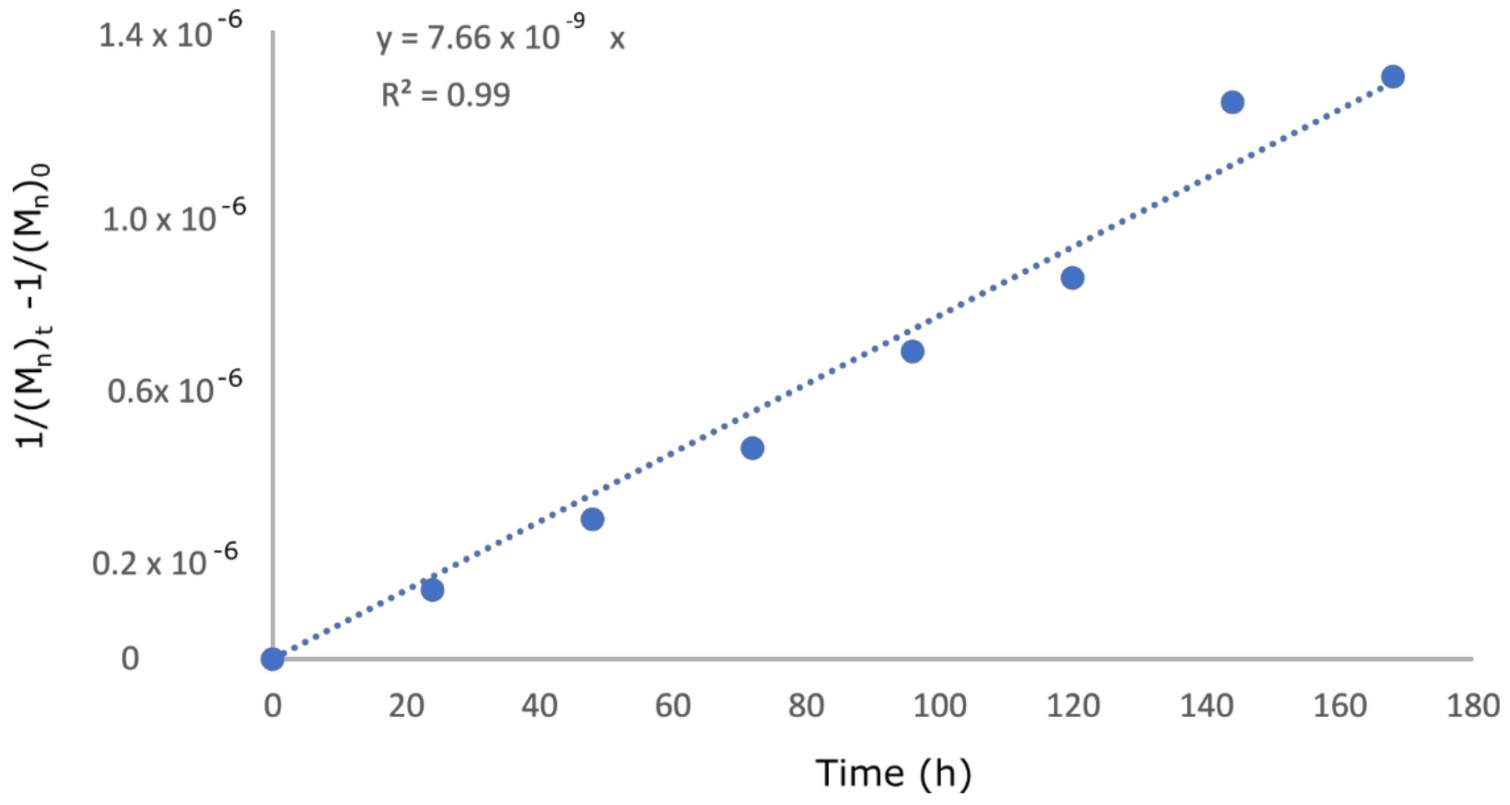
Degradation kinetics Mn values for chitosan in 3% of acetic acid at 20 °C.

**Figure 10:**
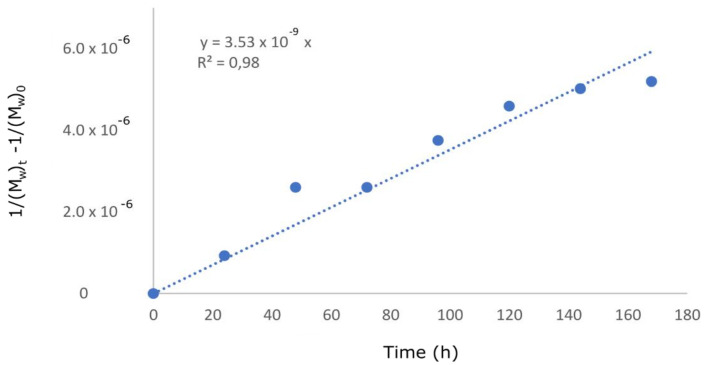
Degradation kinetics Mw values for chitosan in 3% of acetic acid at 20 °C.

Table 6, Table 7 and Table 8 show the value of the constant rate of the chitosan degradation reaction in dilute organic acids, determined on the basis of changes in M_v_, M_n_, and M_w_, respectively. The graphs in Figure 8, Figure 9 and Figure 10 show the kinetics of chitosan degradation in acetic acid for parameters M_v_, M_n_, and M_w_. According to the diagrams, these correspond to the kinetics for first-order and confirm the results from the above tables.

#### H NMR Analysis

**Figure 11:**
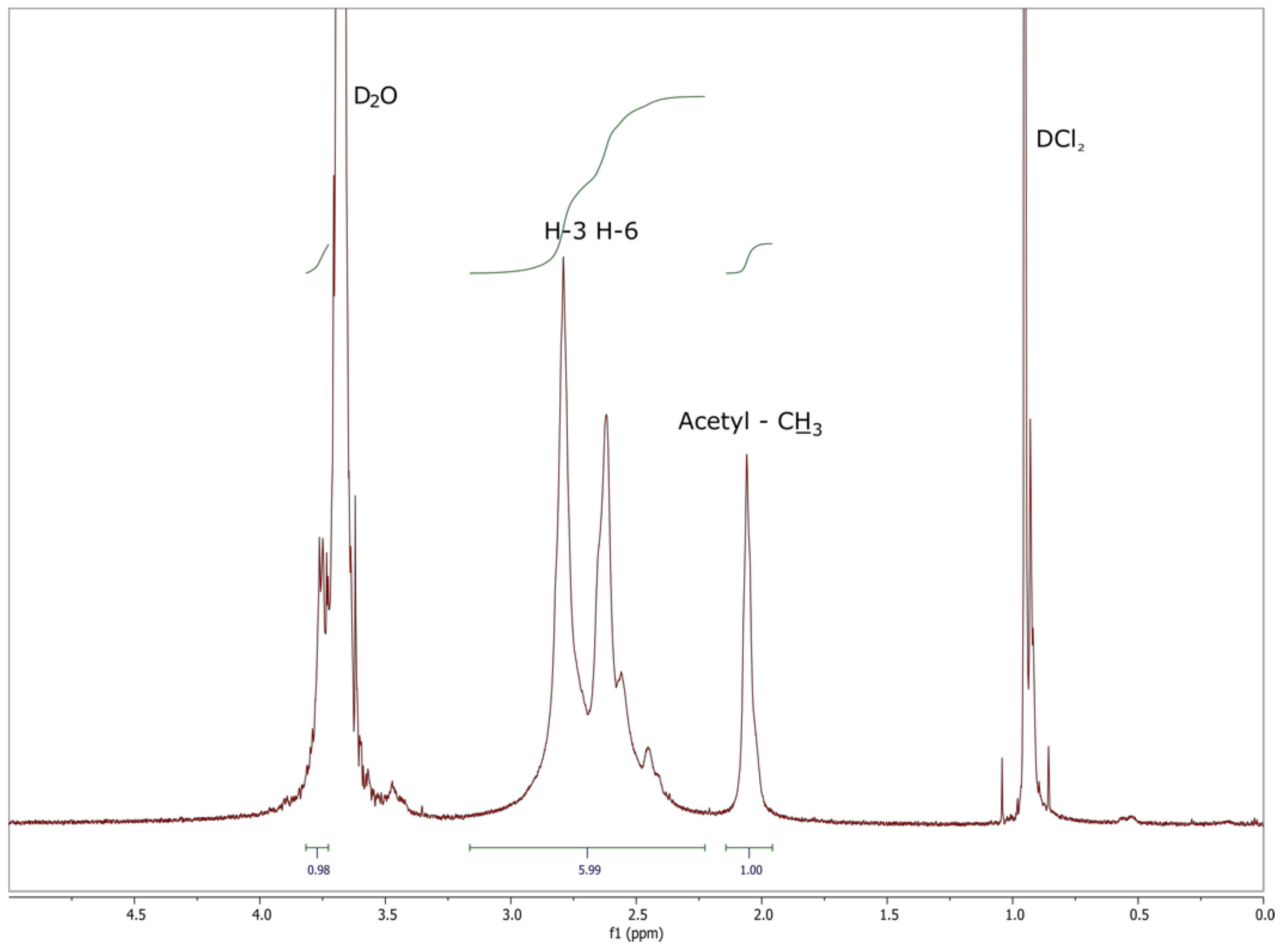
^1^H NMR spectrum of chitosan before degradation (t = 0 min) in diluted acetic acid at 25 °C.

**Figure 12:**
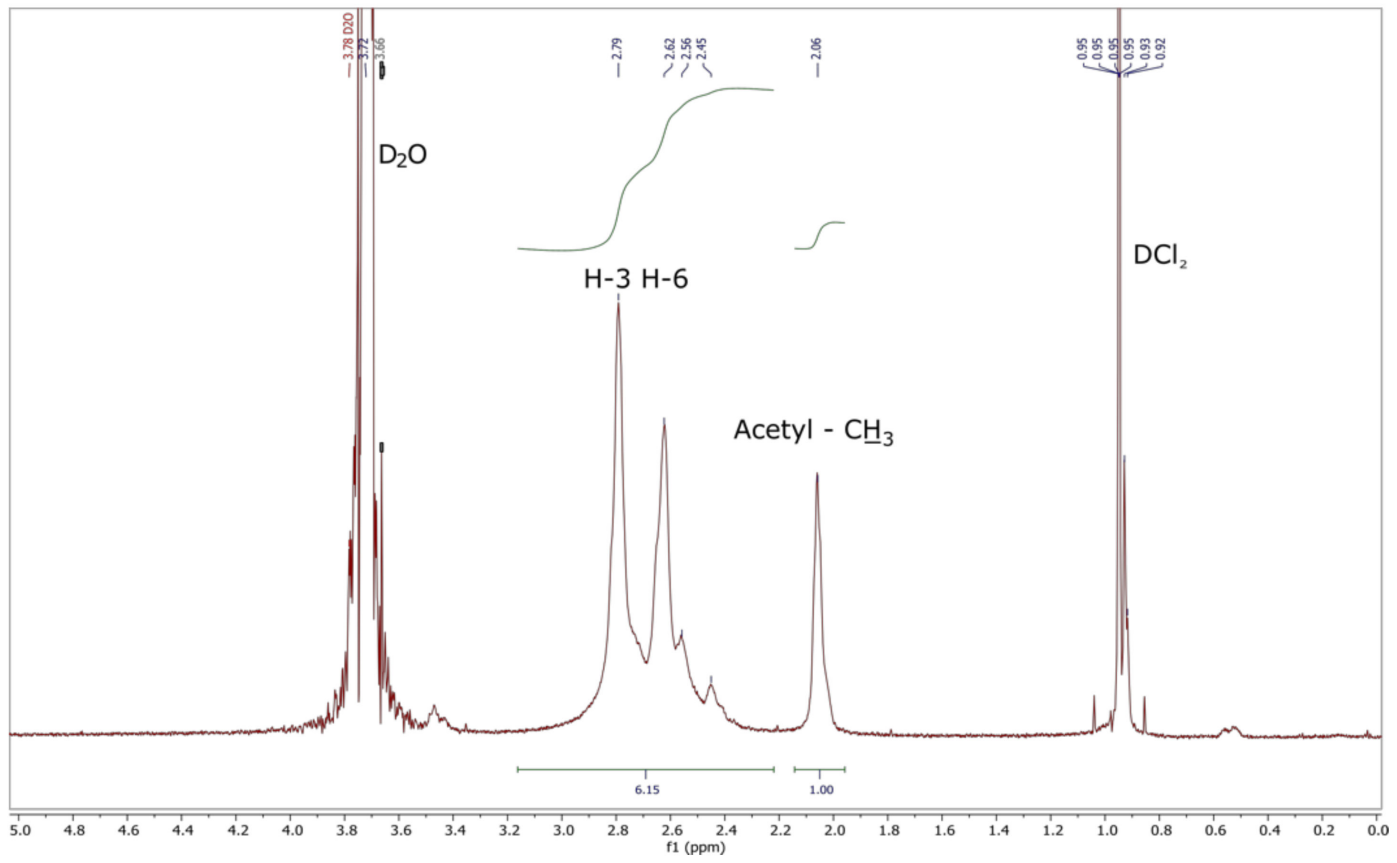
^1^H NMR spectrum of chitosan degraded in diluted acetic acid at 25 °C (t = 168 min).

Figure 11 and Figure 12 show examples of ^1^H NMR spectrum of chitosan before degradation and after 168 h of degradation in acetic acid solution at 20 °C, respectively.

Table 9 shows the results of calculating the degree of deacetylation of chitosan using the formula number (2).

As can be seen from the data summarized in Table 9, the degree of deacetylation does not change significantly, although Figure 11 and Figure 12 certify that the degradation reaction took place. Based on 1H NMR spectra, it can be concluded that the degradation results in the formation of hydroxyl groups at the C1 and C4 positions. The absence of aldehyde groups, characteristic of the degradation of sugars after the breakage of the β-1,4 glycosidic chain at oxidating conditions, confirms that, in organic acid hydrolysis, only shortening of the chitosan polymeric chains takes place.

## 3. Discussion

On the basis of the research carried out in order to determine the intrinsic viscosity and the viscometric average molar mass, it can be concluded that, for chitosan subjected to the action of organic acids, visible changes in the decrease in M_v_ value are observed, indicating the polymer degradation process. The greatest changes in the M_v_ value over time are observed for chitosan treated with malic acid. In the case of formic acid, acetic acid, and lactic acid, the changes in M_v_ are not as rapid in the time as with malic acid. Nevertheless, they also show a destructive effect on chitosan macromolecules. The next acid, in terms of degradation activity, is formic acid, followed by lactic acid, while acetic acid is characterized by the least influence on M_v_ changes over time.

Based on the GPC/SEC analysis, it can be confirmed that the solvents (diluted organic acids) used to prepare chitosan solutions show a destructive effect on the polymer, which results in a decrease in the value of molar masses (M_n_, M_w_) as a function of time. Similar to the results obtained from the measurement of the intrinsic viscosity, the greatest decrease in the value of chitosan molar masses was obtained for malic acid. On the other hand, acetic acid shows the lowest destructive effect on the values of molar masses (M_n_ and M_w_). In the case of GPC/SEC measurements, the results of the analysis indicate that chitosan in malic and lactic acid has the highest decrease in molar mass values during degradation. By analyzing the MMD charts for chitosan after the degradation process in various organic acids, it can be stated that the shape of the distribution of molar masses as a function of time does not change significantly. Based on the analysis of MMD charts, it can be seen that the degradation of chitosan in dilute organic acid solutions is a gradual process. The confirmation of this phenomenon may be the fact that there are no changes in the polydispersity values of chitosan samples taken from a given type of organic acid solution, where values oscillate between 2.27 and 2.74. In the case of degradation in formic, acetic, lactic, and malic acids, no significant changes in the degree of chitosan deacetylation are observed on the basis of ^1^H NMR. The appearance of new proton signals is not observed, which proves the hydrolysis of glycosidic bonds connecting the sugar ring of chitosan. Hydrolysis does not lead to the formation of aldehyde derivatives as is the case with degradation.

## 4. Materials and Methods

Chitosan was purchased as a commercial product Primex-ChitoClear HGQ 110 (Primex, Siglufjordur, Iceland) with a degree of deacetylation of 95% (confirmed by FTIR) and molar mass of 200 kDa. Organic acids, namely, acetic acid (pKa = 4.76), malic acid (pKa = 3.51; 5.03), and formic acid (pKa = 3.75), were commercial products of POCh Gliwice, Poland; lactic acid 80% (pKa = 3.86) was a commercial product of CHEMPUR Piekary Śląskie (Poland). All acids were pure for analysis grade and used without additional purification processes. Distilled water was used to prepare the dilute organic acid solutions.

The procedure for preparing chitosan solutions was as follows: 194 g of water, 6 g of organic acid, and 10 g of chitosan were placed in a conical flask. The prepared solutions were stirred for 24 h with a magnetic stirrer at room temperature; then, the samples were stored in a temperature-controlled chamber at 20 °C.

After the degradation time assumed in the experiment, a part of the solution was taken from the sample subjected to degradation and placed at a temperature of −18 °C in order to stop the further degradation process. The samples were then subjected to a freeze-drying process to remove excess solvent. The obtained porous structures of the chitosan salt and the corresponding acid were dissolved in water. The solutions prepared in this way were samples.

### 4.1. Intrinsic Viscosity Measurements

The procedure for determining the intrinsic viscosity was as follows: samples of chitosan after the degradation process in a suitable solvent in the form of freeze-dried foams were dissolved in 3% acetic acid, obtaining a polymer concentration of 0.2 g/100 cm^3^. Then, an analysis was performed using a viscometer by SI Analytics GMBh, Mainz, Germany, capillary No. 532.11/la at a temperature of 25 °C. Then, a sufficient amount of NaCl was added so that the resulting solutions contained 0.25 M chitosan and 0.25 M sodium acid, respectively. The intrinsic viscosity of chitosan solutions was determined from the Salomon–Ciutâ [49] relationship:

(2) $$\left[\eta \right]=\frac{\sqrt{2}}{c}\sqrt{\eta_{\mathrm{spec}}-\ln\eta_{\mathrm{rel}}\;}$$

where [Ƞ]—intrinsic viscosity; C—polymer concentration; Ƞspec—specific viscosity; Ƞrel—relative viscosity.

The viscometric average molar mass of chitosan was determined according to the Mark–Houwink–Sakrura formula [50] for the parameter K = 1.57 × 10^−4^ and α = 0.79 in accordance with the work [48]:

(3) $$\left[\eta \right]=1.57\times 10^{-4}M_{v}^{0.79}$$

### 4.2. GPC/SEC Analysis

Molecular studies of chitosan, including determination of the molar mass distribution function (MMD), average values of molar masses (M_n_, M_w_), and polydispersity (M_w_/M_n_), were performed using the technic GPC/SEC gel permeation chromatography method. GPC/SEC analysis was performed in a buffer solution containing 0.2 M sodium acetate and 0.3 M acetic acid at a flow rate of 0.8 mL/min. The Agilent Technologies GPC/SEC system with the 1260 ISO Pump and the 1260 ALS autosampler was equipped with an Optilab T-rEX refractometer detector (Wyatt Technology, Goleta, CA, USA). Separation of macromolecules took place in a system of columns (Agilent) 2× PL aquagel OH Mixed, 300 mm long, at 30 °C. Column calibrations were performed using Varian PEO/PEG standards, with molar masses ranging from 1970 D to 278 100 D. The results of the GPC/SEC analysis in the form of the molar mass distribution (MMD) function, mean values of molar masses (M_n_, M_w_), and polydispersity (M_w_/M_n_) were calculated using the universal calibration method, where the parameters a and K of the Mark–Houwink–Sakurada equation are, respectively, for PEO standards/PEG, a = 0.625 and k = 62 × 10^−5^ mL/g, and, for chitosan, a = 0.76 and k = 74 × 10^−5^ mL/g [51].

### 4.3. NMR Spectroscopy

Chemical structure and DDA of initial chitosans, as well as chitosans after acid treatment, were determined by 1H-NMR spectroscopy analysis on Brucker AM 400 spectrometer using a mixed solvent of DCl and D2O (1%, *w/w*), with the presence of DSS as reference. Sample preparation was as follows: 50 mg of chitosan salts, obtained by freeze-drying the solution in a suitable acid, was dispersed in D2O and, after homogenization, dissolved by adding DCl (D2O/DCl 36:1, *v*/*v*). When the solution was formed, measurements were made at 303 K using 32 scans pulse accumulation. The degree of chitosan deacetylation was calculated using the following formula:

(4) $$\mathrm{DAA}=100-\frac{A-{\mathrm{CH}}_{3}}{C3-C6}\times 16.67$$

where A − CH_3_—integral of the area of the acetamide group; C3 − C6—integral of the area of the hydrogen presented at carbons from 3 to 6 at chitosan sugar ring.

### 4.4. Determination of the Kinetics of Chitosan Degradation

The kinetics of chitosan degradation was determined on the basis of the dependence-determining changes in molecular weights as a function of the interaction time of the organic acid. According to the literature data, the kinetic dependence of degradation by hydrolysis is expressed by the formula describing the kinetics according to the first-order mechanism in [51], and the obtained results are confirmed by [52]:

(5) $$\frac{1}{{\left(M_{w}\right)}_{t}}-\frac{1}{{\left(M_{w}\right)}_{t0}}=\frac{k}{M}\times t$$

where (Mw)_0_—average degree of polymerization of the polymer before degradation; (Mw)_t_—average degree of polymerization of the polymer after degradation over the time; k—degradation rate constant; t—degradation time; M—chitosan molar mass.

## 5. Conclusions

The research results presented in this article are of high importance in terms of the stability of polymer processing, where the appropriate parameters of the solutions (M_v_, M_w_, and P_d_) must be maintained for an effective process that remains constant over time. Therefore, identifying the right solution for long industrial processes is an important matter. The search for a suitable solvent for the preparation of a chitosan solution, in which the polymer will not degrade with time, is an especially important issue for the electrospinning process.

The greatest changes in the M_v_ value over time are observed for chitosan treated with malic acid. The analysis of the data obtained from the GPC/SEC show the same result for malic acid. The degradation kinetics of M_v_, M_n_, and Mw confirms the research results obtained in other studies. Due to its chemical structure, with two carboxylic acid groups, malic acid shows a stronger degradation effect on chitosan than other acids with only one carboxyl group, because it exhibits more “hydrophilic character”, which allows easier access to the polymer chain. The result of this is the interaction of the acid carboxyl groups with the hydroxyl groups of chitosan, which leads to the breaking of glycosidic bonds and thus the degradation of chitosan molecules. The presented graphs of kinetics for the values of the parameters M_n_, M_v_, and M_w_ confirm the first-order kinetics for acetic acid solution. No significant changes in the degree of chitosan deacetylation are observed on the basis of ^1^H NMR.

## Figures and Tables

**Table 1:** Results of measurements of the intrinsic viscosity of the chitosan salt subjected to the degradation process and the values of the average viscometric molar mass.

Degradation Time [Hours]	Salt of Malic Acid pKa = 3.51		Salt of Acetic Acid pKa = 4.76	
	η	Mv (g/mol)	η	Mv (g/mol)
0	1.61	119,400	1.58	116,500
24	1.61	119,400	1.57	115,700
48	1.62	118,500	1.55	114,300
72	1.59	117,500	1.54	113,000
96	1.52	111,000	1.53	111,900
120	1.48	107,300	1.52	111,300
144	1.32	106,300	1.51	110,600
168	1.31	106,000	1.51	109,800
**Degradation Time** **[Hours]**	**Salt of Formic Acid** **pKa = 3.75**		**Salt of Lactic Acid** **pKa = 3.81**	
	η	**Mv** **(g/mol)**	η	**Mv** **(g/mol)**
0	1.60	118,500	1.60	118,500
24	1.59	117,500	1.58	116,600
48	1.58	116,600	1.57	115,200
72	1.57	115,700	1.56	114,700
96	1.55	113,800	1.54	112,900
120	1.53	111,900	1.53	111,900
144	1.52	111,000	1.52	111,010
168	1.51	110,100	1.51	110,100

**Table 2:** The value of angular coefficient obtained from linear dependence of M_v_ changes over degradation time.

Acid	pKa	Angular Coefficient Figure 1	Errors for the Angular Coefficients	R^2^
Malic acid	3.51	−150.24	6.14	0.99
Formic acid	3.75	−53.06	2.61	0.98
Lactic acid	3.81	−48.65	2.43	0.98
Acetic acid	4.76	−41.11	2.07	0.98

**Table 3:** Values of molecular parameters determined by GPC/SEC technique.

Degradation Time [Hours]	Salt of Malic Acid pKa = 3.51			Salt of Acetic Acid pKa = 4.76		
	Mn (g/mol)	Mw (g/mol)	Pd (M_w_/_n_)	Mn (g/mol)	Mw (g/mol)	Pd (M_w_/_n_)
0	45,300	114,100	2.52	44,200	114,200	2.58
24	43,800	111,800	2.55	43,900	113,000	2.57
48	43,800	108,100	2.47	43,600	110,900	2.54
72	42,100	106,200	2.52	43,300	110,900	2.56
96	41,400	103,100	2.49	42,900	109,500	2.55
120	40,700	100,500	2.47	42,600	108,500	2.55
144	40,800	100,000	2.45	41,900	108,000	2.58
168	39,650	97,000	2.45	41,800	107,800	2.58
**Degradation Time [Hours]**	**Salt of Formic Acid** **pKa = 3.75**			**Salt of Lactic Acid** **pKa = 3.81**		
	**Mn (g/mol)**	**Mw (g/mol)**	**Pd (M_w_/M_n_)**	**Mn (g/mol)**	**Mw (g/mol)**	**Pd (M_w_/M_n_)**
0	44,600	119,200	2.67	48,500	114,400	2.36
24	44,800	117,000	2.61	46,900	110,000	2.35
48	43,900	116,000	2.64	45,800	108,100	2.36
72	41,900	114,500	2.73	44,800	105,500	2.35
96	41,400	113,400	2.74	44,100	101,400	2.30
120	43,000	112,600	2.62	43,700	101,800	2.33
144	41,700	111,600	2.68	44,100	100,300	2.27
168	41,700	110,000	2.64	42,900	99,000	2.31

**Table 4:** The value of the angular coefficient obtained from linear dependence of Mw changes over the degradation time.

Acid	pKa	Angular Coefficient Figure 6	Errors for the Angular Coefficients	R^2^
Malic acid	3.51	−101.49	5.05	0.99
Formic acid	3.75	−50.94	2.52	0.99
Lactic acid	3.81	−88.94	9.51	0.94
Acetic acid	4.76	−38.89	3.74	0.95

**Table 5:** The value of the angular coefficient obtained from linear dependence of Mn changes over the degradation time.

Acid	pKa	Angular Coefficient Figure 7	Errors for the Angular Coefficients	R^2^
Malic acid	3.51	−32.02	2.98	0.95
Formic acid	3.75	−19.35	5.61	0.66
Lactic acid	3.81	−29.86	4.23	0.89
Acetic acid	4.76	−14.98	0.77	0.98

**Table 6:** Degradation kinetics data developed based on Mv values.

Acid	pKa	k/M (1/h)	Errors for k/M	R^2^
Malic acid	3.51	11.72 × 10^−9^	5.61 × 10^−10^	0.98
Formic acid	3.75	3.78 × 10^−9^	1.30 × 10^−10^	0.99
Lactic acid	3.81	4.01 × 10^−9^	1.06 × 10^−10^	0.99
Acetic acid	4.76	3.27 × 10^−9^	0.75 × 10^−10^	0.99

**Table 7:** Degradation kinetics data developed based on M_n_ values.

Acid	pKa	k/M (1/h)	Errors for k/M	R^2^
Malic acid	3.51	19.26 × 10^−9^	8.51 × 10^−10^	0.99
Formic acid	3.75	9.19 × 10^−9^	4.71 × 10^−10^	0.98
Lactic acid	3.81	17.55 × 10^−9^	1.24 × 10^−9^	0.96
Acetic acid	4.76	7.66 × 10^−9^	2.57 × 10^−10^	0.99

**Table 8:** Degradation kinetics data developed based on Mw values.

Acid	pKa	k/M (1/h)	Errors for k/M	R^2^
Malic acid	3.51	9.23 × 10^−9^	1.93 × 10^−10^	0.99
Formic acid	3.75	4.22 × 10^−9^	1.17 × 10^−10^	0.99
Lactic acid	3.81	9.04 × 10^−9^	4.71 × 10^−10^	0.98
Acetic acid	4.76	3.53 × 10^−9^	1.71 × 10^−10^	0.98

**Table 9:** Calculated based on ^1^H NMR spectra chitosan degree of deacetylation after degradation in diluted organic acids (5%) at a temperature of 20 °C and polymer concentration of 3%.

Sample Name	Degree of Deacetylation (%)	
	0 h	168 h
Acetic acid	72.2	72.9
Lactic acid	72.9	71.6
Formic acid	72.2	72.2
Malic acid	72.2	71.1
